# Predicting Biochemical Recurrence of Prostate Cancer Post-Prostatectomy Using Artificial Intelligence: A Systematic Review

**DOI:** 10.3390/cancers16213596

**Published:** 2024-10-25

**Authors:** Jianliang Liu, Haoyue Zhang, Dixon T. S. Woon, Marlon Perera, Nathan Lawrentschuk

**Affiliations:** 1 E.J. Whitten Prostate Cancer Research Centre, Epworth Healthcare, Melbourne 3002, Australiadixon.woon@unimelb.edu.au (D.T.S.W.);; 2 Department of Urology, The Royal Melbourne Hospital, The University of Melbourne, Melbourne 3052, Australia; 3 Department of Surgery, The University of Melbourne, Melbourne 3052, Australia; 4 Sir Peter MacCallum Department of Oncology, The University of Melbourne, Melbourne 3051, Australia

**Keywords:** artificial intelligence, biochemical recurrence, convolutional neural network, deep learning, machine learning, prostatic neoplasm, prostate cancer

## Abstract

**Background/Objectives**: Biochemical recurrence (BCR) after radical prostatectomy (RP) is a significant predictor of distal metastases and mortality in prostate cancer (PCa) patients. This systematic review aims to evaluate the accuracy of artificial intelligence (AI) in predicting BCR post-RP. **Methods**: Adhering to PRISMA guidelines, a comprehensive literature search was conducted across Medline, Embase, Web of Science, and IEEE Xplore. Studies were included if they utilised AI to predict BCR in patients post-RP. Studies involving patients who underwent radiotherapy or salvage RP were excluded. This systematic review was registered on PROSPERO (International prospective register of systematic reviews) under the ID CRD42023482392. **Results**: After screening 9764 articles, 24 met the inclusion criteria. The included studies involved 27,216 patients, of whom 7267 developed BCR. AI algorithms developed using radiological parameters demonstrated higher predictive accuracy (median AUROC of 0.90) compared to algorithms based solely on pathological variables (median AUROC of 0.74) or clinicopathological variables (median AUROC of 0.81). According to the Prediction Model Risk of Bias Assessment Tool (PROBAST), the overall risk of bias was unclear in three studies due to ambiguous inclusion criteria and the exclusion of many patients because of missing follow-up data. In seven studies, the developed AI outperformed or was at least equivocal to traditional methods of BCR prediction. **Conclusions**: AI shows promise in predicting BCR post-RP, particularly when radiological data were used in its development. However, the significant variability in AI performance and study methodologies highlights the need for larger, standardised prospective studies with external validation prior to clinical application.

## Simple Summary

Biochemical recurrence (BCR) of prostate cancer (PCa) after surgery is marked by an increase in prostate-specific antigen. BCR is associated with the risk of metastatic disease and/or death. This review evaluates the ability of artificial intelligence (AI) to predict BCR in PCa post-operatively. AI demonstrated high accuracy, especially when radiological features are utilised in its development. AI occasionally outperforms traditional methods of BCR prediction. However, due to the limited number of high-quality studies and insufficient external validation, further research is necessary to confirm the reliability and effectiveness of these AI techniques before they can be widely used in clinical practice.

## 1. Introduction

Radical prostatectomy (RP) is one of the main curative treatment options for prostate cancer (PCa). Up to 40% of patients develop biochemical recurrence (BCR) post-RP, which is characterised by elevated levels of prostate-specific antigen (PSA) [1]. BCR is a predictor of the development of distal metastases, PCa-specific mortality, and overall mortality [2,3]. However, there is a lack of consensus regarding the exact numerical cut-off point for defining BCR post-RP. Various tools, including nomograms, have been developed to predict BCR, which often includes risk factors such as the Gleason score, extraprostatic extension (EPE), seminal vesical invasion (SVI), and positive surgical margins (PSM) [4,5,6].

BCR in the absence of radiological and/or histological recurrence of PCa is also termed PSA-only recurrence. The natural history of PSA-only recurrence can be protracted and does not always lead to clinically apparent metastatic disease [7]. Management of PSA-only recurrence remains a dilemma as there is no clear threshold to determine the initiation of salvage treatment to delay the development of metastatic disease [8]. To further complicate matters, it is unclear when to repeat imaging during the management of PSA-only recurrence. Prostate-specific membrane antigen positron emission tomography (PSMA PET) scans have shown potential in detecting early recurrence; however, the likelihood of a positive PSMA PET scan is correlated with the level of PSA elevation [9]. If a PSMA PET scan is done too early, imperceivable metastatic disease may be missed. Existing studies have illustrated the ability of artificial intelligence (AI) to assess intraprostatic cancer and metastatic disease [10,11]. However, there is no review to date examining the role of AI in BCR. This systematic review aims to evaluate the ability of AI in evaluating clinicopathological and radiological factors to predict BCR post-RP.

## 2. Materials and Methods

### 2.1. Literature Search Strategy

This systematic review was registered on PROSPERO (International prospective register of systematic reviews) under the ID CRD42023482392. The Preferred Reporting Items for Systematic Reviews and Meta-Analyses (PRISMA) guidelines was used. A comprehensive literature search was performed on Medline, Embase, Web of Science, and Institute of Electrical and Electronics Engineers (IEEE) Xplore. Key search terms used include artificial intelligence, prostate neoplasm, and biochemical recurrence.

### 2.2. Eligibility Criteria

The eligibility criteria were guided by the population, intervention, comparator, outcome (PICO) framework. The population included all patients who underwent RP and a subset of those who developed BCR post-RP. Studies were excluded if they included patients who developed BCR after radiotherapy, or if RP was performed as salvage therapy. The intervention in question was the use of AI to evaluate clinicopathological and radiological factors to predict BCR post-RP. Examples of clinicopathological factors include age, PSA, digital rectal examination (DRE), family history, prostate biopsy histology, and prostatectomy histology. Radiological modalities could include prostate ultrasound, MRI, and PSMA PET scans. The use of AI for evaluating serum genetic factors was excluded. The predictive capabilities of the AI algorithm may be compared against traditional methods of predicting BCR. Traditional methods of predicting BCR include conventional statistical regression models and validated BCR calculators such as the Cancer of the Prostate Risk Assessment Postsurgical (CAPRA-S) score, Memorial Sloan Kettering Cancer Center (MSKCC) nomogram, or Partin cancer nomogram [12]. The primary endpoint of this systematic review was to evaluate the ability of AI in predicting BCR post-RP.

All English language original research articles published from inception to December 2023 were considered. The following types of studies were excluded: case reports, case series, feasibility studies, reviews, letter to journals, conference abstracts, conference proceedings, and non-peer reviewed articles.

### 2.3. Screening and Study Selection

Title, abstract, and full-text screening were performed independently by two authors (J.L and H.Z), and any unresolved conflicts were resolved by the senior author. The only automation tool used was Covidence (Veritas Health Innovation, Melbourne, VIC, Australia) to assist in the screening process and the removal of duplicate articles.

Data were manually extracted to Excel 2013 (Microsoft Corporation, Redmond, CA, USA). Independent checks of the data were performed by the co-authors. Missing data were reported in the results.

### 2.4. Quality and Risk of Bias Assessment

The quality of each article was evaluated using the 26-item checklist from the Standardized Reporting of Machine Learning Applications in Urology (STREAM-URO) [13]. The STREAM-URO framework aims to ensure the quality of studies, enhance reproducibility and the interpretation of results, and promote engagement with and understanding of machine learning within the urological community. Additionally, the Prediction Model Risk of Bias Assessment Tool (PROBAST) was utilised to evaluate the risk of bias and the applicability of prediction model studies [14]. Assessments using the STREAM-URO and PROBAST checklists were performed independently by two authors (J.L and H.Z), and any unresolved conflicts were resolved by the senior author.

## 3. Results

### 3.1. Screening Process

**Figure 1:**
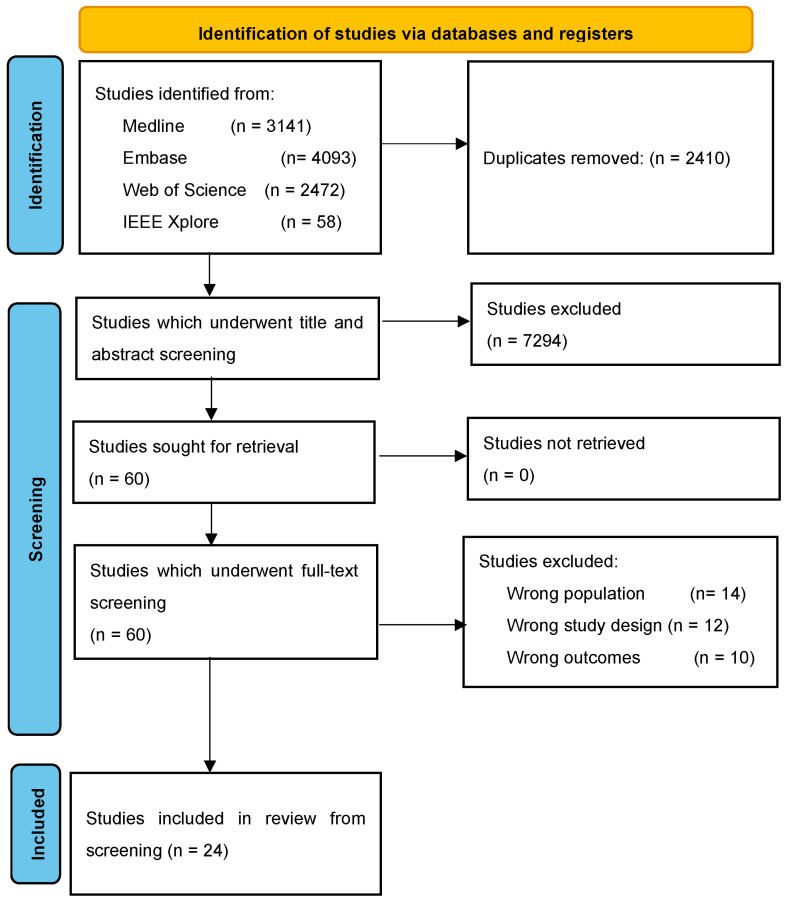
PRISMA flow diagram.

The search yielded 9764 articles, of which 2410 were duplicates (see Figure 1). After the exclusion of 7294 articles during title and abstract screening, 60 studies remained for full-text review. Only 24 studies were included in this systematic review After 36 studies were excluded during full-text review due to inclusion of the wrong study population (n = 14), wrong study design (n = 12), and wrong endpoints (n = 10).

### 3.2. Characteristics of Included Studies

Of the included studies, 21 were retrospective [15,16,17,18,19,20,21,22,23,24,25,26,27,28,29,30,31,32,33,34,35] and 3 were prospective [36,37,38]. The majority of the studies involved only a single centre [17,19,20,23,24,25,26,27,28,30,31,32,33,34,36,37,38], and seven were multicentre [15,16,18,21,22,29,35]. The broad categories of AI techniques used were as follows: machine learning (ML) (n = 2) [18,37], deep learning (DL) (n = 4) [15,16,27,35], neural network (NN) (n = 6) [17,19,25,28,33,34], and random forest classifier (RFC) (n = 1) [22]. The remaining 11 studies used and compared various forms of AI techniques [20,21,23,24,26,29,30,31,32,36,38].

Two studies did not provide a clear definition of BCR [23,33]. The remaining studies used the following definitions of BCR: two consecutive rises in PSA above 0.2 ng/mL post-RP (n = 10) [15,16,17,18,21,27,29,30,31,35,37], any rise in PSA post-RP with a single value above 0.2 ng/mL (n = 8) [19,20,22,26,28,32,36,38], a rise in PSA post-RP above 0.1 ng/mL (n = 2) [24,34], and PSA rise post-RP above 0.3 (n = 1) [25].

### 3.3. Characteristics of Patients in Included Studies

Across the 24 studies, a total of 27,216 patients were included, of which 7267 developed BCR. The median age ranged between 58.9 and 70 years old (not reported in six studies) [15,17,22,26,33,37], and the median PSA before RP ranged between 7 and 13.7 ng/mL (not reported in seven studies) [15,17,18,19,26,33,37]. The median duration of follow-up ranged between 28 and 114 months (not reported in nine studies) [17,20,22,23,26,28,31,33,38], and the median time to BCR was between 11.5 to 48 months (not reported in eighteen studies) [16,17,18,20,21,22,23,24,25,26,27,28,29,31,33,34,37,38].

### 3.4. Quality and Risk of Bias Assessment of Included Studies

**Figure 2:**
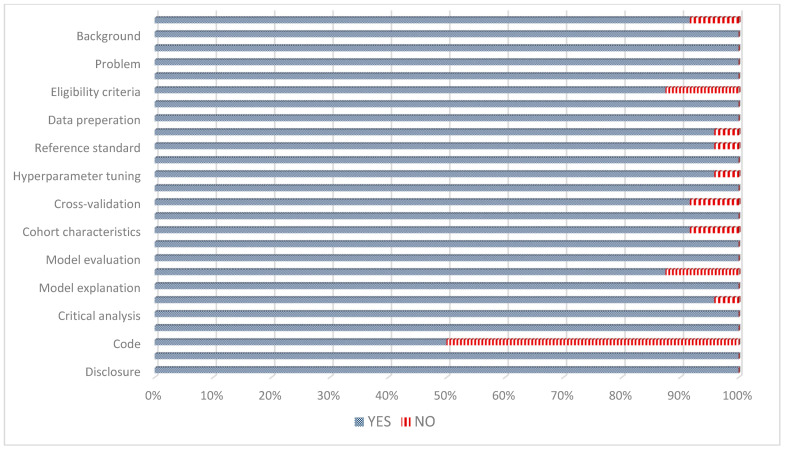
Standardized Reporting of Machine Learning Applications in Urology (STREAM-URO) grading of the included studies.

**Figure 3:**
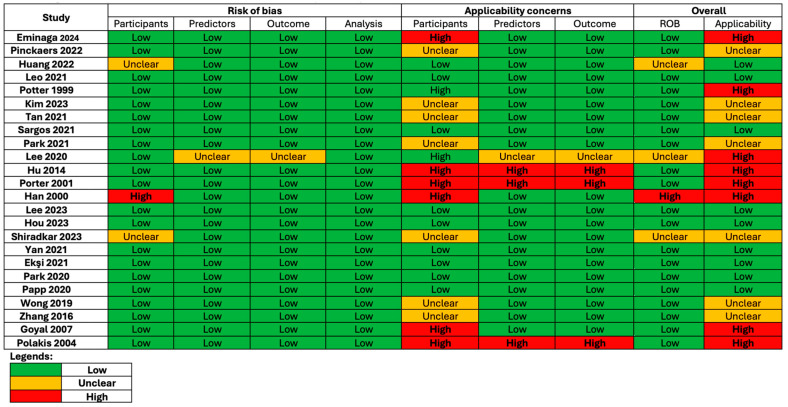
The Prediction Model Risk of Bias Assessment Tool (PROBAST) results for included studies [15,16,17,18,19,20,21,22,23,24,25,26,27,28,29,30,31,32,33,34,35,36,37,38].

The mean STREAM-URO score was 23 out of 26 (See Figure 2). The top three STREAM-URO criteria that were missing were as follows: availability of code used (n = 12) [20,21,22,23,24,25,31,32,33,34,36,38], absence of bias assessment (n = 3) [17,23,33], and absence of eligibility criteria (n = 3) [17,29,33]. On assessment with PROBAST, the overall risk of bias was unclear in three studies due to the following: an absence of explanation regarding the selection of patients for analysis (n = 2) [17,29], or a large amount of follow-up data being unavailable for BCR analysis (n = 1) [26] (See Figure 3). The overall concerns regarding applicability using PROBAST were high in eight studies due to the following: unconventional definitions of BCR (n = 3) [24,25,34], absence of definition of BCR (n = 2) [23,33], only including patients with certain Gleason scores (n = 2) [19,26], and the inclusion of patients with persistent PSA post-RP (n = 1) [15].

### 3.5. AI Developed Using Histological Variables Only

Five of the included studies developed their AI algorithm utilising histological variables only (see Supplementary Table S1). Both Eminaga et al. [15] and Pinckaers et al. [16] developed DL models using RP histology slides to predict BCR. The BCR score in the Eminaga et al. study was closely related to the 10-year BCR-free survival rate on a calibration plot [15]. On external validation using multicentre international data, the DL model developed by Eminaga et al. could predict BCR with an area under the receiver operating characteristic curve (AUROC) of 0.71 (95% CI: 0.67–0.75), sensitivity of 0.5, and specificity of 0.83 [15]. However, it should be noted that the dataset used by Eminaga et al. included patients with persistent PSA post-RP [15]. Although the DL model developed by Pinckaers et al. used a smaller sample of Hematoxylin and Eosin (H&E)-stained microarray cores, the included sample adhered to strict inclusion criteria [16]. Specifically, patients were selected based on two consecutive PSA rises above 0.2 ng/mL after having previously undetectable levels post-radical prostatectomy (RP). Additionally, the patients who experienced BCR were case-matched to those who did not develop BCR. The DL model by Pinckaers et al. was strongly associated with BCR on univariable and multivariable analysis with a Hazard Ratio (HR) of 5.78 (95% CI 2.44–13.72; *p* < 0.005) and 3.02 (CI 1.10–8.29; *p* = 0.03), respectively [16].

Leo et al. [18] developed an ML model using a large multicentre dataset to detect invasive cribriform adenocarcinoma (ICC) on post-RP H&E-stained slides. Patients with a significant amount of ICC had a much higher risk of BCR (HR 1.65, 95% CI 1.13–2.40; *p* = 0.003). The correlation with BCR was strongest in patients with international society of urological pathology grade group (ISUP GG) 2. The association of ICC with BCR was strongest in patients with ISUP GG 2. This ML model may be helpful in identifying patients who may not be suitable for active surveillance.

Huang et al. [17] and Potter et al. [19] developed an NN using single-centre retrospective data. The convolutional NN (CNN) developed by Huang et al. extracted visual and subvisual morphologic features from whole slide images identifying adverse patterns predictive of early recurrence even in low to intermediate ISUP GG PCa [17]. The CNN was able to predict 3-year BCR with an AUROC of 0.78. Potter et al. developed a genetically engineered NN (GENN) using a small sample size involving only patients with a Gleason score of 5 to 7 [19]. Various pathological variables were used to develop this GENN, including the Gleason score, EPE, PSM, nuclear morphometric descriptors (NMDs), and DNA ploidy. The GENN performed the best when developed using NMD and DNA ploidy, with an AUROC of only 0.74, accuracy of 0.8, sensitivity of 0.75, and specificity of 0.85. The GENN outperformed logistic regression (LR) modelling and Cox regression analysis in the prediction of BCR. One of the limitations is that there may have been variations in the reporting of histopathology between pathologists [39].

### 3.6. AI Developed Using Clinical and Histological Variables

Eight of the included studies developed AI algorithms using a combination of clinicopathological inputs (see Supplementary Table S2). These studies used different combinations of age, pre-RP PSA, clinical tumour stage, prostate volume, diagnostic biopsy Gleason score, and prostatectomy histopathology (Gleason score, EPE, SVI, PSM). Five of these studies compared NNs against other types of AI algorithms [20,21,23,24,26]. Han et al. [26] found that an artificial NN (ANN) outperformed LR in 3-year and 5-year BCR prediction with AUROCs of 0.81 and 0.75, respectively. Hu et al. [24] compared an ANN against LR for 10-year BCR prediction and found no statistically significant difference in performance (*p* = 0.53), with AUROCs of 0.75 and 0.76, respectively. It is worth noting that Hu et al. used a lower cut-off for BCR (two consecutive rises in PSA greater than 0.1 ng/mL) [24]. Porter et al. [25] also developed an ANN for 10-year BCR prediction using a smaller sample size and found an AUROC of 0.8, sensitivity of 0.74, and specificity of 0.78.

Kim et al. [20] developed a partial correlation NN (PCNN) which outperformed other types of ML algorithms. The usage of partial correlation also helped improve the interpretability of the NN. The PCNN had an accuracy of 0.87, sensitivity of 0.91, and specificity of 0.86. Sargos et al. [21] compared the 3-year BCR predictive capability of a K-nearest neighbour (KNN), RFC, Cox regression analysis, and a densely connected feed-forward NN (DNN). Sargos et al. [21] found that all models performed best when post-RP variables were added to the models as compared to using CAPRA score variables alone (AUROCs of 0.55, 0.64, 0.64, and 0.7 increased to 0.77, 0.74, 0.75, and 0.84, respectively). Lee et al. [23] utilised data from the Korean Prostate Cancer Registry (KPCR) database and comparatively tested 13 different AI models which included subtypes of RFC, NN, survival regression, and LR. LR performed the best at 5-year BCR prediction, with an AUROC of 0.81, sensitivity of 0.73, and specificity of 0.73.

Tan et al. [36] developed and evaluated three ML models (Naïve Bayes (NB), RFC, and support vector machine (SVM)) using a large sample size. All three ML models showed comparable and strong predictive performance in regard to BCR at 1, 3, and 5 years, with all AUROCs reaching above 0.8. The RFC developed by Park et al. [22] predicted BCR post-RP with an AUROC of 0.99. However, it should be noted that out of the 1130 patients included, only 176 developed BCR.

### 3.7. AI Developed Using Radiological Variables

Eleven of the included articles incorporated radiological variables into the development of their AI algorithm (see Supplementary Table S3). Wong et al. [38] included size of prostate on ultrasound together with 18 other clinicopathological features (such as age, PSA, Gleason score on RP, number of lymph nodes obtained) to develop three AI algorithms (KNN, LR, and RFC). All three AI algorithms outperformed classic Cox regression analysis in predicting 1-year BCR. LR had the highest AUROC when compared to Cox regression analysis (0.98 versus 0.87). The study by Wong et al. was excellent as it compared between various AI techniques and traditional methods of predicting BCR, but the model is only applicable to patients who have early BCR (at 1 year) [38]. Poulakis et al. [34] developed an artificial NN (ANN) using clinicopathological factors together with transrectal ultrasound findings of EPE, SVI, and tumour staging, and achieved an AUROC of 0.77. The performance of the ANN improved to an AUROC of 0.90 when radiological parameters extracted from pelvic MRI were incorporated. It should be noted that Poulakis et al. only included patients who had PSA elevation above 0.1 ng/mL after RP with pelvic lymph node dissection [34].

Four of the studies used MRI parameters to develop and compare different AI algorithms. Ekşi et al. [30] utilised conventional mpMRI parameters (such as prostate volume, PIRADS score, SVI, EPE, and lymph node involvement) from a small sample size to develop their AI. Ekşi et al. demonstrated that their KNN (AUROC of 0.93), LR (AUROC of 0.93), and RFC (AUROC of 0.95) all outperformed classic Cox regression analysis (AUROC of 0.92) in predicting BCR [30]. Park et al. [31] used a small sample size and similar mpMRI parameters to compare four AI algorithms. They found that an auto-encoder (AUROC of 0.64) had the highest predictive ability in terms of 1-year BCR as compared to KNN (AUROC of 0.60), decision tree (AUROC of 0.53), and multilayer perceptron (AUROC of 0.61). Using similar parameters, Zhang et al. [32] developed an SVM (AUROC of 0.96) which outperformed LR (AUROC of 0.89) and D’Amico risk stratification (AUROC of 0.86) in 3-year BCR prediction. Shiradkar et al. [29] extracted prostate shape distension descriptors from MRI to develop an RFC and ML algorithm. Although the sample size was relatively small at 133 patients, a strength of Shiradkar et al.’s study was its comprehensive comparison of various AI algorithms [29]. They found that an integrated model (AUROC of 0.75) performed the best as compared to RFC (AUROC of 0.7) or ML (AUROC of 0.69) alone. Although the integrated model had a higher concordance index (C-index of 0.76) when compared to CAPRA (c-index of 0.69) or Decipher risk (c-index of 0.59), it was only comparable to post-RP CAPRA-S (c-index of 0.75).

One limitation of developing AI models based on traditional MRI parameters is the potential variability in reporting, particularly among less-experienced readers [40]. A potential method for navigating around this is the usage of standardised methods of extracting radiomics from prostate MRI [41]. Two of the studies developed DL algorithms using radiomics extracted from MRI. Lee et al. [27] found that the DL model outperformed the radiomics model in predicting 10-year BCR (AUROC of 0.93 versus 0.68). The DL model developed by Yan et al. [35] performed well in predicting 3-year BCR and 5-year BCR, with AUROCs of 0.84 and 0.83, respectively. Some of the strengths of the study by Yan et al. are the robust sample obtained utilising a multicentre design with strict inclusion criteria requiring two consecutive PSA increases above 0.2 ng/mL, and their minimum follow-up period of three years [35].

Two of the included studies developed distinct types of AI algorithms using MRI. Hou et al. [28] developed a deep survival network (DSN) using MRI radiomics which predicted 3-year BCR with an AUC of 0.79. The BCR definition used in this study was not conventional, and BCR was considered present when there were three consecutive postoperative PSA increases above 0.1 ng/mL over at least 6 weeks, with a final PSA above 0.2 ng/mL, or a single PSA measurement of ≥0.4 ng/mL at least 6 weeks after surgery. Goyal et al. developed a neuro-fuzzy system which predicted BCR well, with a coefficient of correlation of 0.99 [33]. However, the study by Goyal et al. was limited by a sample size of 26 patients [33]. Papp et al. [37] was the only study which utilised PSMA PET/MRI to develop an ML model to predict BCR, and they found an AUROC of 0.90. Although their study was prospective, it was limited by a small sample size.

### 3.8. Comparing AI Models

Eleven of the included studies compared various AI algorithms that were trained on a combination of clinicopathological and radiological data (see Table 1). Kim et al. [20] developed multiple AI algorithms, and the top three best-performing were PCNN, RF, and a tree-based algorithm, with the accuracy of all three models averaging 0.87. Sargos et al. [21] found that the DNN had the best performance in predicting 3-year BCR as compared to KNN, RFC, and Cox regression analysis, with AUROCs of 0.84, 0.77, 0.74, and 0.75, respectively. Lee et al. [23] developed thirteen various AI algorithms, and the top three at predicting 5-year BCR were LR, NN, and RF (AUROCs of 0.81, 0.80, and 0.80, respectively). Park et al. [31] employed a different set of AI algorithms and demonstrated that an auto-encoder outperformed KNN, decision tree, and multilayer perceptron (AUROCs of 0.64, 0.60, 0.53, and 0.61, respectively). The three ML models developed by Tan et al. [36] showed strong predictive performance in terms of BCR at 1, 3, and 5 years, with AUROCs of NB at 0.89, 0.88, and 0.89, RFC at 0.85, 0.88, and 0.89, and SVM at 0.84, 0.85, and 0.86, respectively. In the study by Wong et al. [38], LR appeared to perform the best when compared to RFC and KNN (AUROCs of 0.94, 0.92, 0.90, respectively), whereas the study by Ekşi et al. [30] demonstrated that RFC outperforms KNN and LR (AUROCs of 0.95, 0.93, and 0.93, respectively). In the study by Zhang et al. [32], SVM outperformed LR (AUROCs of 0.96 and 0.89, respectively). Shiradkar et al. [29] demonstrated that an integrated model performed the best as compared to individual algorithms of RFC and ML (AUROCs of 0.75, 0.70, and 0.69, respectively). Hu et al. [24] was one of the few studies that performed statistical comparison between their AI models and found no statistically significant differences between the performance of ANN and LR in 10-year BCR prediction. Interestingly, in the study by Han et al. [26], they found that ANN outperformed LR in 3-year and 5-year BCR prediction. It is worth noting that the study by Han et al. [26] only included patients with Gleason 3+4 or 4+3 prostate cancer. Overall, there was no agreement on which algorithm was superior for predicting BCR.

### 3.9. Comparing AI against Traditional Methods of Predicting BCR

Seven of the included studies compared their AI algorithm against traditional methods of predicting BCR (see Table 1). In the studies by Wong et al. [38] and Ekşi et al. [30], all AI techniques employed (LR, RFC, and KNN) outperformed a conventional statistical regression model. Although all three ML models developed by Tan et al. [36] were equivocal to traditional regression analyses, they outperformed existing nomograms (Kattan, John Hopkins [JHH], CAPSURE) (*p* < 0.001). The ANN developed by Poulakis et al. [34] was comparable to Cox regression analysis and Kattan nomogram in terms of predicting 5-year BCR (AUROCs of 0.77, 0.74, and 0.73, respectively). When pelvic MRI parameters were incorporated into the ANN, it performed significantly better as compared to Cox regression analysis and Kattan nomogram (AUROCs of 0.9, 0.78, and 0.73, respectively). When compared to the CAPRA-S score (C-index range between 0.68 and 0.75), AI appears to outperform in most instances (C-index range between 0.76 and 0.83) [28,29,35]. The DL model (C-index of 0.80) developed by Yan et al. [35] outperformed Gleason grade group systems (C-index of 0.58), the National Comprehensive Cancer Network (NCCN) model (C-index of 0.59), and the CAPRA-S score (C-index of 0.68). The DSN developed by Hou et al. [28] also outperformed the conventional D’Amico score, CAPRA, and CAPRA-S score. Sargos et al. [21] demonstrated that a DNN developed based on CAPRA variables (AUROC of 0.7) outperformed the CAPRA score itself (AUROC of 0.63).

## 4. Discussion

BCR after RP is often the first sign preceding recurrent disease and has been shown to be a predictor of distal metastasis and cancer-specific mortality [3]. Accurately predicting a patient’s risk of BCR post-RP can help with the decision-making process between early adjuvant therapy and a “wait and see” approach [42]. This systematic review highlights the growing role of AI in predicting BCR following RP.

There were two common observations among the studies. Firstly, the integration of radiological parameters improved the AI’s predictive capabilities. The majority of the studies incorporating radiological parameters utilised radiomics extracted from pre-operative prostate MRI scans. These AI algorithms achieved a higher median AUROC of 0.9 as compared to algorithms that were developed based solely on pathological variables (median AUROC of 0.74) or clinicopathological variables (median AUROC of 0.81). Previous research has shown that MRI-based tumour characteristics, such as EPE, SVI, and maximum diameter of index lesion, are predictive of BCR post-RP [43,44]. Radiomics, which involves extracting a large number of complex quantitative features, has also proven to be effective in predicting BCR [45,46]. In one study, the DL algorithm (AUROC of 0.93) was tested against a standard radiomics model (AUROC of 0.68) and demonstrated superior performance [27]. Future studies should continue to integrate radiological parameters with clinicopathological variables during the development of AI models for BCR prediction.

Secondly, in the majority of the studies, the AI algorithms outperformed or at least were equivocal to established PCa risk assessment tools, conventional statistical regression models, and nomograms [21,28,30,34,35,36,37,38]. One of the limitations of traditional BCR prediction tools is the inability to integrate quantitative radiomics. The use of AI offers the advantage of incorporating large amounts of data into predictive models, including MRI-based tumour characteristics and radiomics. This has the potential to improve the accuracy of BCR prediction post-RP.

PSMA PET scans have revolutionised the management of PCa with their high sensitivity and specificity in detecting metastatic PCa [47]. There is growing evidence supporting the use of PSMA PET scans and PSMA-based radiomics for predicting BCR post-RP; in some instances, they outperform clinical prediction models [48,49,50]. However, only one of the included studies developed their AI algorithm using PSMA PET/MRI, demonstrating an AUROC of 0.89 [37]. Further research into this area may be beneficial, as intraprostatic maximum standardised uptake values (SUVmax) have been shown to be prognostic and to correlate with the aggressiveness of PCa [51,52].

Three of the most commonly used models were KNN, LR, and RFC. However, among the eleven included studies that compared AI algorithms, there were no consistent results regarding which algorithm was best for predicting BCR. Theoretically, RFC is the most robust for this task due to its ability to manage complex, non-linear relationships and interactions between a large number of features, making it well suited for datasets with numerous features and diverse types of data. Its ensemble approach, utilising multiple decision trees, provides a strong advantage in generalisation and accuracy. KNN, while useful in scenarios with irregular decision boundaries, can be less effective with complex data and is computationally intensive. LR, though straightforward and interpretable, may not capture complex interactions as effectively as RFC. Despite these theoretical advantages, these three AI algorithms appear to have similar performance [30,38]. No conclusion can be drawn at this stage regarding the best AI algorithm for BCR prediction given the heterogeneity of the studies and the usage of other subtypes of AI algorithms such as auto-encoders and multilayer perceptrons. Further research with more standardised methodologies is needed to identify the most effective approach.

This systematic review was limited by the heterogeneity of the included studies, which precluded a meta-analysis. There were substantial differences in the definitions of BCR, included variables, and outcome measures. Additionally, inclusion criteria were not standardised, with some studies including patients with PSA persistence post-RP [53]. There was also a large variation in endpoints, ranging from 1-year to 10-year BCR predictions, further exacerbating this issue. Given that the mean time to BCR post-RP is approximately 8 years, future studies should consider longer follow-up periods [7]. Additionally, most studies involved small single-centre study cohorts and lacked external validation of their AI algorithms, limiting the generalisability of their findings.

A major limitation of current AI models for BCR prediction is the reliance on post-RP data, such as prostatectomy histopathology, which limits their pre-operative utility. Additionally, many AI models suffer from the “black box” issue, where their decision-making processes are not transparent [54]. This lack of transparency complicates the clinical utility of AI, as clinicians cannot provide clear explanations of how predictions are made, which hinders their ability to offer meaningful information about patient risk and the rationale behind treatment recommendations.

To improve the accuracy and clinical applicability of AI models for predicting BCR, future studies could focus on several areas. Firstly, ensemble AI approaches which combine multiple AI algorithms could improve performance by reducing bias and variance [55]. This was demonstrated in the study by Shiradkar et al. [29]. Additionally, the incorporation of a more explainable AI algorithm such as RFC or DT may help navigate the “black box” issue [56]. Secondly, a standardised definition of BCR should be used. The definition endorsed by the American Urological Association (AUA), European Association of Urology (EAU), and National Comprehensive Cancer Network (NCCN) is two consecutive rises in PSA above 0.2 ng/mL post-RP [57,58,59]. Lastly, larger prospective studies with long-term follow-up and external validation are needed to ensure more generalisable results.

## 5. Conclusions

In conclusion, current AI models have shown promising results for predicting BCR post-RP, particularly when imaging modalities such as MRI are incorporated during development. In many cases, these models outperformed or were at least equivocal to traditional methods of BCR prediction. However, the current AI models are not ready for real-life clinical application due to the lack of high-quality prospective studies with robust external validation.

## Figures and Tables

**Table 1:** Included studies which compared AI models or compared them against traditional methods of BCR prediction.

Author and Year	Data Input	AI Models and/or Traditional Methods of BCR Prediction Used	Findings
Kim 2023 [20]	Clinicopathological variables	PCNN vs. SVM vs. RFC	Top three best-performing were PCNN, RF, and a tree-based algorithm, with the accuracy of all three models averaging 0.87.
Lee 2020 [23]	Clinicopathological variables	RFC vs. NN vs. LR vs. decision tree vs. gradient boosting classifier	Top three at predicting 5-year BCR were LR, NN, and RF (AUROCs of 0.81, 0.80, and 0.80, respectively).
Hu 2014 [24]	Clinicopathological variables	ANN vs. LR	The AUROCs of ANN (0.75) and LR (0.76) outperformed the Gleason score (0.71) and T-stage or PSA (0.62) in predicting 10-year BCR.
Han 2000 [26]	Clinicopathological variables	ANN vs. LR	The ANN outperformed LR in predicting 3-year BCR with an AUROC of 0.81 versus 0.68.
Park 2020 [31]	Clinicopathological variables and MRI	KNN vs. MLP vs. DT vs. auto-encoder	Auto-encoder showed the highest prediction ability in 1-year BCR after RP (AUC = 0.638), followed by MLP (AUC = 0.61), KNN (AUC = 0.60), and DT (AUC = 0.53).
Zhang 2016 [32]	Clinicopathological variables and MRI	SVM vs. LR	When compared to LR, SVM had significantly higher AUROC (0.96 vs. 0.89; *p* =0.007), sensitivity (93.3% vs. 83.3%; *p* = 0.025), specificity (91.7% vs. 77.2%; *p* =0.009), and accuracy (92.2% vs. 79.0%; *p* = 0.006) in predicting 3-year BCR.
Wong 2019 [38]	Clinicopathological variables, prostate ultrasound size, and operative variables	KNN vs. RFC vs. LR vs. conventional statistical regression model	KNN, RFC, and LR outperformed the conventional statistical regression model in predicting 1-year BCR. Respectively, the AUCs were 0.90, 0.92, and 0.94, and the accuracy values were 0.98, 0.95, and 0.98.
Ekşi 2021 [30]	Clinicopathological variables and mpMRI	RFC vs. KNN vs. LR vs. conventional statistical regression model	All ML models outperformed the conventional statistical regression model in the prediction of BCR. The AUROCs for RFC, KNN, and LR were 0.95, 0.93, and 0.93, respectively.
Tan 2021 [36]	Clinicopathological variables	Naive Bayes vs. RFC vs. SVM vs. traditional regression analyses vs. nomograms	AUCs for the prediction of BCR at 1, 3, and 5 years for Naive Bayes were 0.894, 0.876, and 0.894, for RFC were 0.846, 0.875, and 0.888, and for SVM were 0.835, 0.850, and 0.855, respectively. Although all three ML models were equivocal to traditional regression analyses, they outperformed existing nomograms (Kattan, John Hopkins [JHH], CAPSURE).
Sargos 2021 [21]	Clinicopathological variables	KNN vs. RFC vs. DNN vs. CAPRA score	The DNN model showed the highest AUC, 0.84, in predicting 3-year BCR when compared to LR, KNN, RF, and Cox regression, with AUC values of 0.77, 0.58, 0.74, and 0.75, respectively. The DNN developed based on CAPRA variables (AUROC of 0.7) outperformed the CAPRA score itself (AUROC of 0.63).
Hou 2023 [28]	Clinicopathological variables and mpMRI radiomics	Deep survival network vs. CAPRA score	The deep survival network could match a histopathological model (Concordance index 0.81 to 0.83 vs. 0.79 to 0.81, *p* > 0.05) and has a maximally 5.16-fold, 12.8-fold, and 2.09-fold (*p* < 0.05) benefit compared to the conventional D’Amico score, the CAPRA score, and the CAPRA Postsurgical score.
Shiradkar 2023 [29]	Biparametric MRI	RFC and ML vs. CAPRA score	Integration of RFC and ML performed the best at predicting BCR, with an AUC of 0.75 as compared to random forest classifier (0.70, *p* = 0.04) or ML (0.69, *p* = 0.01) alone.
Yan 2021 [35]	Quantitative features of MRI	DL vs. CAPRA score vs. NCCN model vs. Gleason grade group systems	The DL model (C-index of 0.80) developed outperformed Gleason grade group systems (C-index of 0.58), NCCN model (C-index of 0.59), and the CAPRA-S score (C-index of 0.68).
Poulakis 2004 [34]	clinicopathological variables, ultrasound, and MRI	ANN vs. Cox regression analysis vs. Kattan nomogram	ANN was comparable to Cox regression analysis and Kattan nomogram in terms of predicting 5-year BCR (AUROCs of 0.77, 0.74, and 0.73, respectively). With the addition of MRI findings, ANN outperformed Cox regression and Kattan nomogram, with an AUC of 0.897, in predicting 5-year BCR.

Abbreviations: ANN (Artificial Neural Network), AUROC (Area Under the Receiver Operating Characteristic Curve), BCR (Biochemical Recurrence), CAPRA (Cancer of the Prostate Risk Assessment), DNN (Deep Neural Network), DT (Decision Tree), DL (Deep Learning), KNN (K-Nearest Neighbor), LR (Logistic Regression), ML (Machine Learning), MLP (Multilayer Perceptron), NCCN (National Comprehensive Cancer Network), NN (Neural Network), PCNN (Probabilistic Convolutional Neural Network), PSA (Prostate-Specific Antigen), RFC (Random Forest Classifier), RP (Radical Prostatectomy), SVM (Support Vector Machine).

## Data Availability

The data presented in this study are available in this article.

## Supplementary Materials

The following supporting information can be downloaded at: https://www.mdpi.com/article/10.3390/cancers16213596/s1, Table S1: Characteristics and results of studies developed using histological variables only; Table S2: Characteristics and results of studies developed using clinical and pathological variables only; Table S3: Characteristics and results of studies that included radiological parameters. References [15,16,17,18,19,20,21,22,23,24,25,26,27,28,29,30,31,32,33,34,35,36,37,38] are cited in the Supplementary Materials.
