# Supplementary material for: Predicting Biochemical Recurrence of Prostate Cancer Post-Prostatectomy Using Artificial Intelligence: A Systematic Review
Source: Cancers (Basel). 2024 Oct 25;16(21):3596. doi: 10.3390/cancers16213596 (PMC11545810; doi:10.3390/cancers16213596)
Supplement: Supplementary file 1 [file cancers-16-03596-s001.zip › cancers-3208246-SI.pdf]

Supplementary Table S1. Characteristics and results of studies developed using histological variables only.

Author and year	Definition of BCR	AI model and study design	Inclusion criteria	Sample size	Data input	Findings	Strengths	Limitations
Eminaga 2024 [15]	2 consecutive PSA ≥ 0.2 ng/mL<br><br>Or failure of PSA to fall < 0.1 ng/mL<br><br>Or initiation of salvage<br><br>Or cancer-specific death	DL<br><br>Multicentre<br><br>Retrospective	**Inclusion**<br>All post-RP patients	1,489 of which 493 had BCR	Histopathology slide	Ability to predict BCR with AUROC 0.71 (95% CI: 0.67–0.75), sensitivity 0.5, specificity 0.83	Multicentre and international dataset<br><br>10-year follow-up	Included patients with persistent PSA post-RP
Pinckaers 2022 [16]	2 consecutive PSA > 0.2 ng/mL after a previously undetectable level after prostatectomy)	DL<br><br>Multicentre<br><br>Retrospective	**Inclusion**<br>Patients who develop BCR post-RP<br>**Exclusion**<br>insufficient tumour amount in the cores	889 of which 386 had BCR	Tissue in H&E-stained microarray cores	On external validation cohort, the DL system predicted recurrence with hazard ratio of 5.78 (95% CI 2.44–13.72; p < 0.005)	Case matching to non-BCR patients with similar histology	Included patients with local recurrence and distal metastasis

	Or BCR events (local recurrence, metastases, PCa-related death)							
Huang 2022 [17]	2 consecutive PSA > 0.2 ng/ mL	CNN<br><br>Single centre<br><br>Retrospective	**Inclusion**<br>Patients from The Cancer Genome Atlas Prostatic Adenocarcinoma (TCGA-PRAD)	416 of which 170 had BCR	Histopathology slide	AUC predicting 3-years BCR was 0.78<br><br>AUC of predicting recurrence in ISUP GG 1, 2, 3 was 0.76, 0.84, and 0.81 respectively.	Ability to predict early recurrence, even in PCa with low or intermediate ISUP GG	Small sample size
Leo 2021 [18]	2 consecutive PSA > 0.2 ng/ mL	ML<br><br>Multicentre<br><br>Retrospective	**Inclusion**<br>successfully digitized H&E slide,<br>PSA <0.2 ng/ml after surgery,<br>**Exclusion**<br>history of neoadjuvant or adjuvant therapy.	819 of which 242 had BCR	Histopathology slide	ML was able to identify cribriform area index which was associated with BCR (concordance index of 0.62)	Large sample size and multi-center	Variation in grading of histology slides

Potter 1999 [19]	PSA > 0.2 ng/mL	GENN<br><br>Single centre<br><br>Retrospective	**Inclusion**<br>RP for Gleason score of 5 to 7 and clinical Stage T1b-T2c cancer<br>**Exclusion**<br>Inadequate follow up or incomplete data	214 of which 84 had BCR	Histological variables	GENN developed using nuclear morphometric descriptors and DNA Ploidy could predict 5-year BCR with AUROC of 0.74, accuracy of 0.80, specificity of 0.85, and sensitivity of 0.75	Trialled various combination of data input	Only applicable to Gleason score 5-7 patients
Abbreviations = Area under the curves (AUCs), Area under the receiver operating characteristic curve (AUROC), Biochemical recurrence (BCR), Confidence interval (CI), Convolutional neural network (CNN), Deep learning (DL), Genetically engineered neural network (GENN), Hema-toxylin and eosin (H&E), Machine learning (ML), rostate cancer (PCa), Radical prostatectomy (RP)								

Supplementary Table S2. Characteristics and results of studies developed using clinical and pathological variables only.

Author and year	Definition of BCR	AI model and study design	Inclusion criteria	Sample size	Data input	Findings	Strengths	Limitations
Kim 2023 [20]	Nadir PSA ≥ 0.2 ng/mL	PCNN vs SVM vs RFC<br>Single centre<br>Retrospective	**Inclusion**<br>All post-RARP patients<br>**Exclusion**<br>Did not have at least 60 months or have missing data	1,021 of which 283 had BCR	Clinicopathological variables	PCNN achieved higher performance than other ML techniques in predicting 5-year BCR with accuracy of 0.87, sensitivity of 0.91, and specificity of 0.86	Used partial correlation to avoid “black box” of neural network	Only a single PSA reading considered BCR
Tan 2021 [36]	PSA > 0.2 ng/ mL	Naive Bayes vs RFC vs SVM<br>Single centre<br>Prospective	**Inclusion**<br>All RARP patients<br>**Exclusion**<br>Persistent PSA > 0.05 ng/ml postoperatively. Patients who had received hormonal therapy or immediate adjuvant radiotherapy.	1,130 of which 176 had BCR	Clinicopathological variables	AUC for prediction of BCR at 1, 3, and 5 years of Naive Bayes was 0.894, 0.876, and 0.894, RF was 0.846, 0.875, and 0.888, and SVM was 0.835, 0.850, and 0.855, respectively	Large sample size and long follow up	Small proportion of patient had BCR
Sargos 2021 [21]	2 consecutive PSA ≥ 0.2 ng/mL	KNN vs RFC vs DNN<br>Multicentre	**Inclusion**<br>Patients who underwent RP for clinically localized<br>**Exclusion**	4,246 of which 817 had BCR	Clinicopathological variables	DNN model shows the highest AUC of 0.84 at predicting 3-year BCR when compared to LR, KNN, RF, and cox regression with AUC	Compared between different statistical and AI techniques	Based on patient who underwent ultrasound

	or additional treatment > 6 months after RP	Retrospective	Neoadjuvant/adjuvant hormone therapy or radiation therapy. Patients with <12months follow up.			value of 0.77, 0.58, 0.74 and 0.75, respectively		guided biopsy
Park 2021 [22]	PSA > 0.2 ng/mL at any point after RP	RFC<br><br>Multicentre<br><br>Retrospective	**<u>Inclusion</u>**<br>Patients who underwent RP<br>**<u>Exclusion</u>**<br>Patients with missing data on follow-up and T-stage	6,755 of which 2,200 had BCR	Clinicopathological variables	RFC predicted BCR post-RP with accuracy of 0.97 and AUROC of 0.99	Used several ensemble sampling technique to account for data imbalance	Broad definition of BCR
Lee 2020 [23]	Not defined	RFC vs NN vs LR vs decision tree vs gradient boosting classifier<br><br>Multicentre<br><br>Retrospective	**<u>Inclusion</u>**<br>Koreans who underwent RP<br>**<u>Exclusion</u>**<br>Neoadjuvant therapy, foreigners, and less than 12 months follow up	5,114 of which 1,207 had BCR	Clinicopathological variables	LR performed the best in predicting 5-year BCR with AUC of 0.81, accuracy of 0.73, sensitivity of 0.73, specificity of 0.73	Trialled and compared various AI techniques	BCR not defined

Hu 2014 [24]	2 consecutive PSA > 0.1 ng/mL	ANN vs LR<br><br>Single centre<br><br>Retrospective	**<u>Inclusion</u>**<br>Patients who underwent laparoscopic RP	1,575 of which 275 had BCR	Clinicopathological variables	The AUROC of ANN (0.75) and LR (0.76) outperformed Gleason score (0.71) and T-stage or PSA (0.62) at predicting 10-year BCR	Trialled and compared various AI techniques	Low cut off for BCR
Porter 2001 [25]	PSA > 0.3 ng/mL<br><br>Or initiation of adjuvant therapy	ANN<br><br>Single centre<br><br>Retrospective	**<u>Inclusion</u>**<br>Patients who underwent RP<br>**<u>Exclusion</u>**<br>Incomplete data	175 of which 77 had BCR	Preoperative clinicopathological variables	ANN predicts PSA failure with AUROC 0.80, sensitivity of 0.74, specificity of 0.78, PPV of 0.71, and a NPV of 0.81	Single pathologist reviewed all specimens allows for consistency	Small sample size<br><br>Mean follow up of 2.5 years
Han 2000 [26]	PSA > 0.2 ng/mL	ANN vs LR<br><br>Single centre<br><br>Retrospective	**<u>Inclusion</u>**<br>Patients who underwent RP with Gleason score 3+4 or 4+3<br>**<u>Exclusion</u>**<br>Adjuvant therapy before BCR	452 of which 130 had BCR	Clinicopathological variables	The ANN outperformed LR in predicting 3-year BCR with AUROC of 0.81 versus 0.68	Trialled and compared various AI techniques	Small sample size

Abbreviations = Area under the curves (AUCs), Area under the receiver operating characteristic curve (AUROC), Artificial neural network (ANN), Biochemical recurrence (BCR), Densely connected feed-forward neural network (DNN), K-Nearest Neighbor (KNN), Logistic regression (LR), Negative predictive value (NPV), Partial correlation neural network (PCNN), Positive predictive value (PPV), Prostate cancer (PCa), Radical prostatectomy (RP), Robotic assisted radical prostatectomy (RARP), Random forest classifier (RFC), Support vector machine (SVM)

Supplementary Table S3. Characteristics and results of studies that included radiological parameters.

Author and year	Definition of BCR	AI model and study design	Inclusion criteria	Sample size	Data input	Findings	Strengths	Limitations
Lee 2023 [27]	2 consecutive PSA ≥ 0.2 ng/mL	DL<br><br>Single centre<br><br>Retrospective	**<u>Inclusion</u>**<br>Patients who underwent RP after 3T multiparametric MRI (mpMRI)<br>**<u>Exclusion</u>**<br>Poor-quality MRI<br>PSA persistence postoperatively	437 of which 110 had BCR	Clinicopathological variables and mpMRI radiomics	DL combining clinical variables and mpMRI radiomics performed the best as compared to either input alone.<br><br>iAUC of 0.93<br><br>HR of 7.72 (95% CI: 1.24–14.19, p = 0.0008)	Trialled multiple variables in development of DL	Excluded patients with persistent PSA post-RP<br><br>Some patients underwent PLND<br><br>Unclear spilt of training/ validation/ test data
Hou 2023 [28]	3 consecutive increasing PSA values > 0.1 ng/ml at least 6 weeks with	Deep survival network<br><br>Single centre<br><br>Retrospective	**<u>Inclusion</u>**<br>All post-RP patients	579 of which 171 had BCR	Clinicopathological variables and mpMRI radiomics	The deep survival network (iBCR-Net) can match a histopathological model (Concordance index 0.81 to 0.83 vs 0.79 to 0.81, p > 0.05); and has maximally 5.16-fold,	Uses stepwise processing operations integrating radiomics, predicting T3	Median follow-up of 26.1 months

	final PSA > 0.2 ng/ml<br><br>OR PSA ≥ 0.4 ng/ml once at least 6 weeks post-operatively					12.8-fold, and 2.09-fold (p < 0.05) benefit to conventional D'Amico score, the CAPRA score and the CAPRA Postsurgical score.	stage and lymph node metastasis.	
Shiradkar 2023 [29]	2 consecutive PSA ≥ 0.2 ng/mL	RFC and ML<br><br>Multicentre<br><br>Retrospective	**Inclusion**<br>All post-RP patient who had 3T MRI prior and follow up for ≥3 years	133 of which 39 had BCR	Biparametric MRI	Integration of RFC and ML performed the best at predicting BCR with AUC 0.75 as compared to random forest classifier (0.70, p = 0.04) or ML (0.69, p = 0.01) alone.	Trialled and compared various AI techniques	Small sample size
Yan 2021 [35]	2 consecutive PSA > 0.2 ng/mL	DL<br><br>Multicentre<br><br>Retrospective	**Inclusion**<br>Patients who underwent RP<br>**Exclusion**<br>ADT pre-op, less than 3 years follow-up, persistent PSA post-RP	485 of which 146 had BCR	Quantitative features of MRI	The DL achieved concordance index of 0.802 outperforming the CAPRA-S score (0.677), NCCN model (0.586) and Gleason grade group systems (0.583).	Multicentre with minimum of 3 years follow up	Small sample size

Ekşi 2021 [30]	2 consecutive PSA > 0.2 ng/mL	RFC vs KNN vs LR<br><br>Single centre<br><br>Retrospective	**Inclusion**<br>Patients who underwent RARP<br>**Exclusion**<br>history of chemotherapy, radiotherapy, auto-immune disease, steroid use, and infections during the preoperative assessment	368 of which 73 had BCR	Clinicopathological variables and mpMRI	All ML models out- performed the conventional statistical regression model in the prediction of BCR. The AUROC for RFC, KNN, and LR were 0.95, 0.93, and 0.93, respectively	Trialled and compared various AI techniques	Small sample size
Park 2020 [31]	2 consecutive PSA > 0.2 ng/mL	KNN vs MLP vs DT vs auto-encoder<br><br>Single centre<br><br>Retrospective	**Inclusion**<br>Patients who underwent MRI and RP<br>**Exclusion**<br>Previous hormone therapy, and poor quality of MRI	104 of which 20 had BCR	Clinicopathological variables and MRI	Auto-encoder showed the highest prediction ability of 1-year BCR after RP (AUC = 0.638), followed by MLP (AUC = 0.61), KNN (AUC = 0.60), and DT (AUC = 0.53)	Trialled and compared various AI techniques	Small sample size
Papp 2020 [37]	2 consecutive PSA ≥ 0.2 ng/mL	ML<br><br>Single centre	**Inclusion**<br>Patients who underwent RP<br>**Exclusion**	36 of which 9 had BCR	Radiological features (PSMA PET/MRI and T2w and ADC of MRI)	ML predicted 5-year BCR post-RP with accuracy of 0.98 and AUROC of 0.89	Prospective data	Small sample size

		Prospective	Missing BCR data					
Wong 2019 [38]	PSA > 0.2 ng/mL at 1 year follow-up	KNN vs RFC vs LR<br><br>Single centre<br><br>Prospective	**Inclusion**<br>Patients who underwent RARP	338 of which 25 had BCR	Clinicopathological variables, prostate ultrasound size, and operative variables	KNN, RFC, and LR outperformed conventional statistical regression model at predicting 1-year BCR. Respectively the AUC were 0.90, 0.92, 0.94 and the accuracy were 0.98, 0.95, and 0.98	Trialled and compared various AI techniques	Only applicable to early BCR
Zhang 2016 [32]	PSA ≥ 0.2 ng/mL	SVM vs LR<br><br>Single centre<br><br>Retrospective	**Inclusion**<br>Patients who underwent MRI and RP<br>**Exclusion**<br>Adjuvant radiation or hormone therapy prior to BCR, MRI performed externally	205 of which 61 had BCR	Clinicopathological variables and MRI	When compared to LR, SVM had significantly higher AUROC (0.96 vs. 0.89; p = 0.007), sensitivity (93.3% vs. 83.3%; p = 0.025), specificity (91.7% vs. 77.2%; p = 0.009) and accuracy (92.2% vs. 79.0%; p = 0.006) for predicting 3-year BCR	Trialled and compared various AI techniques and different combination of data input	Small sample size
Goyal 2007 [33]	Not specified	Neuro-fuzzy<br><br>Single centre	**Inclusion**<br>Patients who underwent open retropubic RP	26	Preoperative clinicopathological variables and MRI	Coefficient of correlation was 0.9935	Comprehensive pre-operative factors used to include family	Small sample size

		Retrospective	**Exclusion**<br>Patients lost to follow up				history and DRE	Only 6 months follow up
Poulakis 2004 [34]	PSA > 0.1 ng/mL	ANN<br><br>Single centre<br><br>Retrospective	**Inclusion**<br>Patients who underwent RP with PLND<br>**Exclusion**<br>Immediate post-op hormone or radiation therapy	210 of which 73 had BCR	clinicopathological variables, ultrasound, and MRI	With addition of MRI findings, ANN outperformed Cox regression and Kattan nomogram with AUC 0.897 at predicting 5-year BCR	Trialled multiple variables in development of ANN	Specific for patients who underwent PLND
Abbreviations = Apparent diffusion coefficient (ADC), Area under the curves (AUCs), Area under the receiver operating characteristic curve (AUROC), Artificial neural network (ANN), Biochemical recurrence (BCR), Cancer of the Prostate Risk Assessment post-surgery (CAPRA-S), Deep learning (DL), K-Nearest Neighbor (KNN), Logistic regression (LR), Machine learning (ML), Magnetic resonance imaging (MRI), Multilayer perceptron (MLP), Pelvic lymph node dissection (PLND), Prostate cancer (PCa), Radical prostatectomy (RP), Robotic assisted radical prostatectomy (RARP), Random forest classifier (RFC), Support vector machine (SVM)								
